# Self-Esteem, Lover-Centeredness, and Social Networking Site Use as Mediators Between Resilience and Cyberbullying Victimization

**DOI:** 10.1007/s11126-025-10154-6

**Published:** 2025-05-13

**Authors:** M. Furkan Kurnaz, Nilüfer Koçtürk

**Affiliations:** 1 Department of Guidance and Psychological Counseling, Necmettin Erbakan University, Konya, Türkiye; 2 Department of Guidance and Psychological Counseling, Hacettepe University, Ankara, Türkiye

**Keywords:** Cyberbullying victimization, Resilience, Self-esteem, Social networking site use, Lover-centeredness, Serial mediation model

## Abstract

Individual factors play a crucial role in shaping the strength of the relationship between resilience and cyberbullying victimization. This study examines the mediating roles of self-esteem, lover-centeredness, and social networking site use in this relationship through a serial mediation model. A total of 597 adults (460 females, mean age: 22.25) participated in the study, meeting G*Power’s sample size recommendations. Preliminary analyses were conducted using SPSS 27.0 and JASP, while the serial mediation model was tested using the SPSS PROCESS Macro. Additionally, a multi-group analysis was performed using SmartPLS 3 to explore differences in the serial mediation model based on participants’ relationship status. The findings indicated that resilience does not significantly predict cyberbullying victimization. However, self-esteem and social networking site use were found to mediate this relationship both individually and jointly, whereas lover-centeredness did not emerge as a significant mediator. Moreover, self-esteem, lover-centeredness, and social networking site use demonstrated a significant combined mediating effect on the relationship between resilience and cyberbullying victimization. The multi-group analysis revealed that resilience significantly predicts lover-centeredness among individuals in a romantic relationship, but this prediction was not significant for those without a lover. These results offer valuable insights into the interplay between resilience, intimate relationships, and cyberbullying victimization.

## Introduction

Online social networking sites allow individuals to connect with others who share similar interests, effectively addressing limitations in traditional forms of communication [1]. While the frequent use of social networking sites by individuals in romantic relationships can facilitate communication, enhance emotional bonds, and provide avenues for expressing feelings [2], it has also been linked to an increase in cyberbullying victimization [3]. According to Cava et al. [4], individuals’ perceptions of their lovers are associated with cyber dating violence. These perceptions, particularly those related to lover-centeredness, are rooted in the context of romantic love, often conceptualized as a “tolerance for everything through love” [5].

Self-esteem, which reflects an individual’s emotional state and degree of integration within interpersonal relationships [6], may play a critical role in shaping lover-centeredness. This is because self-esteem encompasses a wide range of self-beliefs, including self-assessments of emotions, beliefs, and behaviors [7]. Self-esteem is directly linked to resilience, which is defined as a set of developable traits that enable individuals to endure adverse and stressful life experiences, overcome challenges, and demonstrate better-than-expected outcomes [8]. In this context, resilience helps individuals become less affected by cyberbullying victimization, thereby protecting their overall well-being [9]. Recent large-scale syntheses (e.g., [10]) have confirmed that low resilience and self-esteem are among the most consistent psychological predictors of cyberbullying victimization across diverse populations and settings.

To integrate these constructs into a unified model, this study employs Ecological Systems Theory [11] as its guiding framework. This theory emphasizes the dynamic interplay between individuals and their environments at multiple levels, ranging from personal traits (e.g., resilience and self-esteem) to immediate social contexts (e.g., romantic relationships and interactions on social networking sites) [12]. Applying this framework allows us to examine how internal characteristics and external relational influences jointly contribute to the experience of cyberbullying victimization [13]. Specifically, this study explores how self-esteem, lover-centeredness, and social networking site use as mediators in the relationship between resilience and cyberbullying victimization, thereby providing a more integrated understanding of how personal and relational factors interact to influence individuals’ vulnerability to cyberbullying on social networking sites.

## Theoretical Frameworks

### Cyberbullying

Cyberbullying is defined as “an aggressive act that is repeated, intentional, and performed within a specific time period against a victim who is unable to defend themselves well, using electronic forms of communication by a group or individual” [14], p. 376). Common examples of cyberbullying include sending harmful messages or posting inappropriate images of others [15]. Previous studies have linked cyberbullying to suicidal thoughts [16], anxiety [17], and depression [18]. According to the Cyberbullying Research Center [19], one in four children experiences cyberbullying victimization, while approximately one in six engages in cyberbullying perpetration. A systematic review on adults [20] reported that the prevalence of cyberbullying victimization ranged from 2.38% to 90.86%, while the prevalence of perpetration ranged from 0.56% to 54.3%.

### Social Networking Site Use

The rise of cyberbullying has coincided with the increasing reliance on digital technologies as primary communication tools [21, 22]. This phenomenon is particularly prevalent with the widespread use of social networking sites [23]. Social networking sites are websites and applications that allow users to share thoughts, content, beliefs, emotions, opinions, and personal and social experiences [24]. Research has established links between excessive social networking site use and negative psychological outcomes, including anxiety [25], depression [26], and loneliness [27].

### Lover-Centeredness

Lover-centeredness refers to lover-oriented behaviors characterized by a willingness to sacrifice unconditionally for the presence of a lover or the continuation of the relationship [28]. While this construct shares certain features with existing concepts such as romantic obsession, emotional dependency, and anxious attachment, it is distinct in both scope and emphasis. Romantic obsession often involves intrusive thoughts and compulsive behaviors toward a lover [29] and anxious attachment reflects a deep-seated fear of abandonment and hyperactivation of attachment needs [30]. In contrast, lover-centeredness emphasizes the internalization of cultural and relational ideals, such as dedication, loyalty, and self-sacrifice, which guide individuals’ behavior in romantic relationships, often at the expense of personal well-being or autonomy.

The construct integrates elements from various relationship frameworks, including interdependence theory [31], which emphasizes the mutual influence lovers exert on one another’s outcomes, and attachment theory [32], which underscores the emotional bonds and dependency patterns in close relationships. By situating lover-centeredness within these frameworks, it is possible to conceptualize it as an interactional phenomenon that reflects both cognitive schemas and behavioral tendencies aimed at maintaining relational stability and security [28].

This concept also incorporates beliefs such as the idea that love has the power to resolve all difficulties, that love is essential for happiness, that jealousy signifies love, that love is inherently tied to suffering, and that a soulmate is the only true form of love [33]. Individuals with high levels of lover-centeredness may be less likely to recognize cyberbullying victimization, as they may interpret such experiences as expressions of genuine affection [34].

### Self-Esteem

Self-esteem reflects the degree of interaction an individual has within interpersonal relationships and serves as a subjective measure of their connection to society and significant others [6]. It encompasses a person’s attitude toward themselves, including their self-love and self-respect [35]. Research indicates that individuals with high self-esteem experience lower levels of depression [36] and substance use [37], greater emotional regulation [38], higher life satisfaction [39], and less frequent use of social networking sites [40].

### Resilience

Resilience is defined as the ability to adapt positively to significant threats by utilizing personal resources, maintaining a constructive outlook, and drawing on environmental support [41]. It strengthens individuals’ capacity to manage adversity, thereby promoting their overall well-being [42]. Collen and Onan [9] found that resilience reduces the negative psychological impact of cyberbullying and prevents deterioration in mental health. Similarly, Peker and Yalçın [43] demonstrated that resilience weakens the positive correlation between social networking site use and cyberbullying.

### Current Study

Although numerous studies on cyberbullying exist in the literature, many aspects remain underexplored, highlighting the need for further research as cyberbullying continues to be a significant phenomenon [44]. Specifically, the increasing instances of cyberbullying victimization resulting from the misuse of social networking sites warrant closer investigation [24]. Within this context, lover-centeredness may serve as a critical factor influencing social networking site use and subsequent cyberbullying victimization. Conversely, self-esteem, which shapes individuals’ perceptions in interpersonal relationships [6], may act as a protective factor against lover-centeredness, excessive social networking site use, and cyberbullying victimization [41]. Resilience, which incorporates foundational elements of self-esteem [45–47], could play a significant role as a protective factor by enhancing individuals’ ability to adapt to and cope with cyberbullying victimization.

**Fig. 1:**
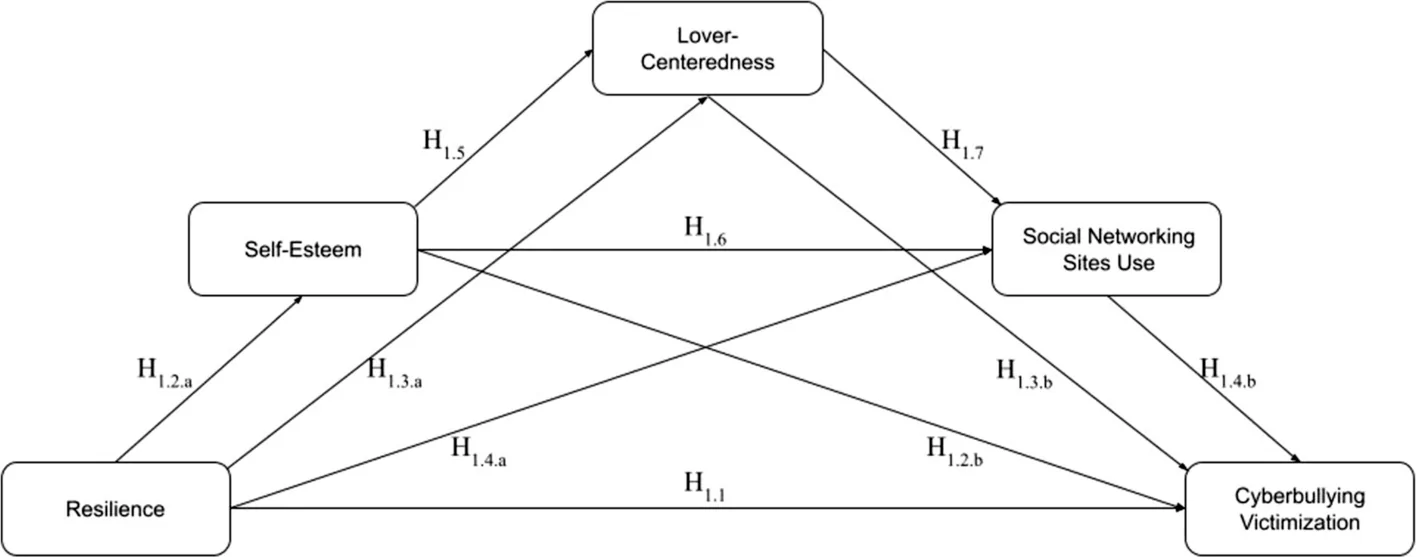
Proposed mediating model

However, a review of the existing literature reveals a lack of studies examining the indirect roles of social networking site use, lover-centeredness, and self-esteem in the relationship between resilience and cyberbullying victimization. To address this gap, the current study aims to explore the mediating roles of self-esteem, lover-centeredness, and social networking site use in this relationship. Building on previous research, the following hypotheses, illustrated in Fig. 1, were developed:

- H1. Self-esteem, lover-centeredness, and social networking site use mediate the relationship between resilience and cyberbullying victimization.
- H1.1. Resilience significantly predicts cyberbullying victimization.
- H1.2. Self-esteem mediates the relationship between resilience and cyberbullying victimization.
- H1.2.a. Resilience significantly predicts self-esteem.
- H1.2.b. Self-esteem significantly predicts cyberbullying victimization.
- H1.3. Lover-centeredness mediates the relationship between resilience and cyberbullying victimization.
- H1.3.a. Resilience significantly predicts lover-centeredness.
- H1.3.b. Lover-centeredness significantly predicts cyberbullying victimization.
- H1.4. Social networking site use mediates the relationship between resilience and cyberbullying victimization.
- H1.4.a. Resilience significantly predicts social networking site use.
- H1.4.b. Social networking site use significantly predicts cyberbullying victimization.
- H1.5. Self-esteem significantly predicts lover-centeredness.
- H1.6. Self-esteem significantly predicts social networking site use.
- H1.7. Lover-centeredness significantly predicts social networking site use.
- H2. The direct effects in the serial mediation model vary based on whether individuals on have a lover.

## Method

### Participants and Procedure

G*Power was used to determine the necessary sample size for testing the mediation model. Following Faul et al.’s [48] recommendations, the analysis, based on four predictors, an effect size of 0.02, and a statistical power of 0.80, indicated a required minimum of 602 participants. A total of 703 individuals participated between May 16 and May 29, 2024, after obtaining ethical approval from the [masked] University Ethical Board (Report Number: [masked]). Outliers were identified using Mahalanobis distances at a 0.05 significance level, resulting in the removal of 106 data points. The remaining 597 participants ensured an effect size of 0.02 and a statistical power of 0.796, deemed sufficient for the mediation model analysis [48].

The final sample consisted of 597 participants, of whom 460 (77.1%) were female and 137 (22.9%) were male, with an age range of 18 to 58 years (*M* = 22.25, *SD* = 4.39). Relationship status varied, with 57.3% (*n* = 340) reporting no current partner, 27.8% (*n* = 166) having a partner, 6.9% (*n* = 41) dating, 6% (*n* = 36) married, and 2% (*n* = 12) engaged.

### Data Collection Tools

#### Short-Form of the State Self-Esteem Scale (SSES-S)

The SSES-S, developed by Brito et al. [49] and adapted into Turkish by Kurnaz and Koçtürk [50], contains 10 items across three factors: academic performance, physical appearance, and social success. The Turkish form demonstrated high internal consistency (ω = 0.90,α = 0.80; λ6 = 0.89) and good fit indices [*X*^2^ (32) = 126.57; *p* <. 001; CFI = 0.96; GFI = 0.99; TLI = 0.95; RMSEA = 0.08, (90% CI: 0.07 to 0.10); SRMR = 0.07]. In this study, internal consistency values were ω = 0.68, α  = 0.76 and λ6 = 0.85.

#### Revised Cyber Bullying Inventory II (RCBI- II)

The RCBI-II, revised by [51], assesses cyberbullying levels. Among participants aged 13–21, the scale exhibited good fit indices (GFI = 0.93, AGFI = 0.89, CFI = 0.93, NFI = 0.89, TLI = 0.90; RMSEA = 0.06). Sub-dimension reliability scores were α = 0.82 for the cyberbullying perpetration and α = 0.75 for victimization. In this study, the victimization sub-dimension showed McDonald’s ω = 0.85, Cronbach’s α = 0.84, and Guttman λ6 = 86.

#### Social Networks Sites Use

Developed by Topaloglu et al. [52], this scale measures social networking site usage with 13 items and two sub-dimensions: “aims of use” and “social networking site use.” Reliability scores for the overall scale were α = 0.88, with the sub-dimensions scores of α = 0.87 and α = 0.86. This study used the “social networking site use” sub-dimension, which showed reliability values of ω = 0.78, α = 0.78, λ6 = 0.77.

#### Brief Resilience Scale

The Brief Resilience Scale [53], adapted into Turkish by Doğan [54], demonstrated good fit indices during the adaptation process (*X*^2^ = 12.86; *df* = 7; NFI = 0.99; NNFI = 0.99; CFI = 0.99; RMSEA = 0.05; IFI = 0.99; TLI = 0.98; RFI = 0.97; AGFI = 0.96; SRMR = 0.03). The Turkish version had a Cronbach’s α of 0.76. In this study, internal consistency values were ω = 0.78, α = 0.78, and λ6 = 0.79.

#### Lover-Centeredness Scale (LCS)

The LCS, developed by [28], was designed to assess beliefs, emotions, and behaviors that reflect the prioritization of a romantic partner in one’s life. The scale is grounded in theoretical frameworks such as interdependence theory [31] and attachment theory [32], capturing relational dynamics characterized by idealization, emotional intensity, and self-sacrifice. Conceptually, lover-centeredness refers to the internalization of cultural narratives that promote loyalty, devotion, and the belief that love can overcome all difficulties, often leading individuals to prioritize their lover’s needs above their own.

The item pool for the LCS was initially developed through a comprehensive literature review on romantic beliefs, emotional dependency, and relational idealization. The preliminary items were reviewed by experts in relationship psychology for content validity and clarity. The finalized version of the scale consists of nine items across three sub-dimensions: perceptions of the lover’s existence, dedication to the lover, and value attributed to the lover. Sample items include statements such as “Not having a lover shows I am a failure.” and “I would give up everything for my lover’s happiness.” Items are rated on a five-point Likert scale ranging from 1 (strongly disagree) to 5 (strongly agree), with higher scores indicating stronger lover-centeredness.

The scale demonstrated high reliability (ω = 0.94; α = 0.94; λ6 = 0.95) and good fit indices based on confirmatory factor analysis [*X*^2^ (24) = 133.95 *p* < 0.001; CFI = 0.93; GFI = 0.92; TLI = 0.90; RMSEA = 0.15, (90% CI: 0.13 to 0.18); SRMR = 0.04]. In the current study, the reliability values were ω = 0.86, α = 0.86, and λ6 = 0.90.

## Data Analysis

Descriptive statistics, including missing data identification, were performed using SPSS 27.0. Internal consistency analyses were conducted using JASP, while CR and HTMT values were assessed via SmartPLS 3. The measurement model was evaluated with SPSS Amos 24. To examine the mediating roles of self-esteem, lover-centeredness, and social networking site use in the relationship between resilience and cyberbullying victimization, the SPSS PROCESS Macro (Model 6; [55]) was utilized.

## Results

### Preliminary Analyses

A frequency analysis revealed no missing data in the dataset. The means, standard deviations, skewness, and kurtosis values of all items were examined. Skewness values ranged from –0.07 to 1.90, and kurtosis values ranged from –0.62 to 4.34, indicating that the data followed a normal distribution [56]. Detailed descriptive statistics for the study variables are presented in Table 1.

**Table 1:** Descriptive statistics and correlations of variables in serial mediation model

Variables	*M*	*SD*	*S*	*K*	1	2	3	4	5
R	19.13	4.25	0.08	0.10	–	.51^***^	–.10^*^	–.24^***^	–.13^**^
SE	36.48	6.34	–0.07	–0.51		–	–.13^**^	–.27^***^	–.18^***^
LC	13.81	4.80	0.71	–0.41			–	.15^***^	.10^*^
SNSU	13.85	4.82	0.26	–0.62				–	.18^***^
CV	14.02	4.83	1.90	4.34					–

*N* = 597, *R* Resilience, *SE* Self-Esteem, *LC* Lover-Centeredness, *SNSU* Social Networking Site Use, *CV* Cyberbullying Victimization, ^***^p <.001; ^**^p <.01; ^*^p <.05; *M* Mean, *SD* Standard Deviation, *S* Skewness, *K* Kurtosis

Internal consistency coefficients for all variables exceeded 0.68, indicating acceptable reliability [57, 58]. Composite reliability (CR) values ranged from 0.82 to 0.90, demonstrating high reliability [59]. Multicollinearity was assessed, with tolerance values (TV) ranging from 0.71 to 0.97, variance inflation factor (VIF) values ranging from 1.03 to 1.40, and a Durbin-Watson value of 1.99, suggesting no significant multicollinearity [59]. Discriminant validity was confirmed using Heterotrait-Monotrait (HTMT) ratios, with all values below 0.90 [60]. Multicollinearity, reliability, and validity findings are summarized in Table 2.

**Table 2:** Multicollinearity, validity, and reliability

Variables	*MC*		Reliability				Validity (HTMT)				
	TV	VIF	ω	α	λ6	*CR*	1	2	3	4	5
R	0.73	1.37	.78	.78	.79	.85	–	.64	.13	.31	.17
SE	0.71	1.40	.68	.76	.85	.82		–	.19	.35	.22
LC	0.97	1.03	.86	.86	.90	.90			–	.20	.14
SNSU	0.90	1.11	.78	.78	.77	.84				–	.21
CV	–	–	.85	.84	.86	.89					–

*R* Resilience, *SE* Self-Esteem, *LC* Lover-Centeredness, *SNSU* Social Networking Site Use *CV* Cyberbullying Victimization *MC* Multicollinearity *TV* Tolerance Value, *VIF* Variance Inflation Factor, ω McDonald's Omega, α Cronbach's Alpha, α6 Guttman's Lambda6, *CR* Composite Reliability, *HTMT* Heterotrait-Monotrait Ratio

Pearson correlation analysis showed that resilience positively correlated with self-esteem (*r* = 0.51, *p* < 0.001) and negatively correlated with lover-centeredness (*r* = –0.10, *p* = 0.017), social networking site use (*r* = –0.24, *p* < 0.001), and cyberbullying victimization (*r* = –0.13, *p* = 0.002). Self-esteem negatively correlated with lover-centeredness (*r* = –0.13, *p* = 0.002), social networking site use (*r* = –0.27, *p* < 0.001), and cyberbullying victimization (*r* = –0.18, *p* < 0.001). Lover-centeredness positively correlated with social networking site use (*r* = 0.15, *p* < 0.001) and cyberbullying victimization (*r* = 0.10, *p* = 0.016). Additionally, social networking site use positively correlated with cyberbullying victimization (*r* = 0.18, *p* < 0.001).

### Measurement Model

The measurement model was assessed for suitability in the mediation model. Items for social networking site use and resilience were parceled [61, 62]. The model demonstrated an acceptable fit: [*X*^2^(55, *N* = 597) = 159.17, *X*^2^/*df* = 2.89; RMSEA = 0.056; SRMR = 0.068; CFI: 0.95; GFI: 0.96; IFI: 0.95]. All factor loadings were above 0.40 and statistically significant.

### Serial Mediation Model

After validating the measurement model, the mediating roles of self-esteem, lover-centeredness, and social networking site use in the relationship between resilience and cyberbullying victimization were examined using the SPSS PROCESS Macro (Model 6). Bootstrap sampling (5,000 iterations) with a 95% bias-corrected confidence interval (CI) was employed, with statistical significance determined if the CI did not include zero [55].

**Fig. 2:**
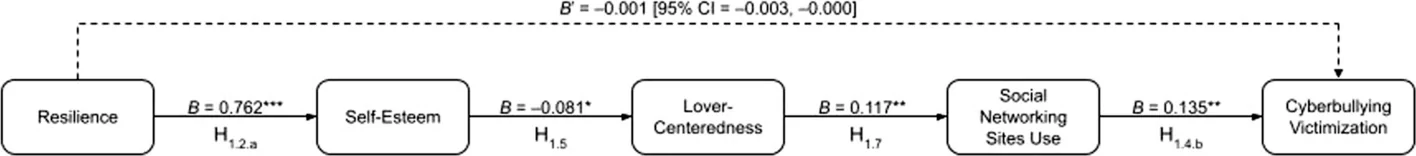
Direct effects in mediating model

As shown in Table 3 and Fig. 2, resilience did not significantly predict cyberbullying victimization (*B* = –0.028, *SE* = 0.053, %95 CI [–0.133, 0.076]). However, resilience significantly predicted self-esteem (*B* = 0.762, *SE* = 0.053, 95% CI [0.659, 0.865]) and social networking site use (*B* = –0.147, *SE* = 0.051, 95% CI [–0.248, –0.046]) but not lover-centeredness (*B* = –0.048, *SE* = 0.053, 95% CI [–0.153, 0.057]). Self-esteem significantly predicted lover-centeredness (*B* = –0.081, *SE* = 0.036, 95% CI [–0.151, –0.011]), social networking site use (*B* = –0.141, *SE* = 0.035, 95% CI [–0.209, –0.073]), and cyberbullying victimization (*B* = –0.097, *SE* = 0.036, 95% CI [–0.168, –0.026]). While lover-centeredness significantly predicted social networking site use (*B* = 0.117, *SE* = 0.040, 95% CI [0.039, 0.194]), it did not significantly predict cyberbullying victimization (*B* = 0.059, *SE* = 0.041, 95% CI [–0.021, 0.140]). Social networking site use significantly predicted cyberbullying victimization (*B* = 0.135, *SE* = 0.042, 95% CI [0.052, 0.218]).

**Table 3:** Regression-based results in serial mediation model

H	DV	IV	*R*	*R*^2^_adj_	*F*	*B*	*t*	95% CI
H_1.2.a_	SE	R	0.511	0.260	210.13	0.762^***^	14.50	0.659, 0.865
H_1.3.a_	LC	R	0.134	0.015	5.43	–0.048	–0.90	–0.153, 0.057
H_1.5_		SE				–0.081^*^	–2.26	–0.151, –0.011
H_1.4.a_	SNSU	R	0.313	0.094	21.51	–0.147^**^	–2.86	–0.248, –0.046
H_1.6_		SE				–0.141^***^	–4.08	–0.209, –0.073
H_1.7_		LC				0.117^**^	2.95	0.039, 0.194
H_1.1_	CV	R	0.238	0.050	8.91	–0.028	–0.53	–0.133, 0.076
H_1.2.b_		SE				–0.097^**^	–2.69	–0.168, –0.026
H_1.3.b_		LC				0.059	1.45	–0.021, 0.140
H_1.4.b_		SNSU				0.135^**^	3.19	0.052, 0.218

*DV* Dependent Variable, *IV* Independent Variable, *R* Resilience, *SE* Self-Esteem, *LC* Lover-Centeredness, *SNSU* Social Networking Site Use, *CV* Cyberbullying Victimization, *H* Hypotheses, *B* Unstandardized Beta, *CI* Confidence Interval

The total effect of resilience on cyberbullying victimization was significant [*B* = –0.145, *SE* = 0.046, 95% CI (–0.236, –0.054)]. Self-esteem significantly mediated the relationship between resilience and cyberbullying victimization [*B* = –0.074, *SE* = 0.029, 95% CI (–0.134, –0.019)]. However, lover-centeredness did not mediate this relationship [*B* = –0.003, *SE* = 0.004, 95% CI (–0.012, 0.003)]. Social networking site use significantly mediated the relationship [*B* = –0.020, *SE* = 0.010, 95% CI (–0.041, –0.004)]. The combined mediating effects of self-esteem and social networking site use were significant (*B* = –0.015, *SE* = 0.006, 95% CI [–0.028, –0.004]), whereas other combinations were insignificant. Full details are presented in Table 4.

**Table 4:** Indirect and total predictions

Hypothesis	Predict	*B*	*SE*	Bootstrapping 95% CI
H_1.2_	R – SE – CV	–0.074	0.02	–0.134, –0.019
H_1.3_	R – LC – CV	–0.003	0.00	–0.012, 0.003
H_1.4_	R – SNSU – CV	–0.020	0.01	–0.041, –0.004
	R – SE – LC – CV	–0.004	0.00	–0.011, 0.001
	R – SE – SNSU – CV	–0.015	0.01	–0.028, –0.004
	R – LC – SNSU – CV	–0.001	0.00	–0.003, 0.001
	R – SE – LC – SNSU – CV	–0.001	0.00	–0.003, –0.000
H_1_	Total Indirect Prediction	–0.116	0.03	–0.180, –0.059
H_1.1_	Direct Prediction	–0.028	0.05	–0.133, 0.076
	Total Prediction	–0.145	0.05	–0.236, –0.054

*R* Resilience, *SE* Self-Esteem, *LC* Lover-Centeredness, *SNSU* Social Networking Site Use, *CV* Cyberbullying Victimization, *B* Unstandardized Beta, *SE* Standard Error, *CI* Confidence Interval

#### Multi-Group Analysis Based on Having a Lover

A multi-group analysis was conducted using SmartPLS 3 to examine differences based on relationship status (H2). Participants were divided into those without a lover (*n* = 342; female = 268; *M* = 21.36, *SD* = 2.62) and those with a lover (*n* = 255; female = 192; *M* = 23.44, *SD* = 5.79).

As shown in Table 5, resilience significantly predicted lover-centeredness for participants with a lover (*ß* = –0.188, *SD* = 0.07, 95% CI [–0.327, –0.039]), whereas this prediction was insignificant for participants without a lover (*ß* = 0.037, *SD* = 0.07, 95% CI [–0.095, 0.159]).

**Table 5:** Multi-group analysis results based having a lover

Path	*ß*		*t*		Predict Dif	*p*
	No	Yes	No	Yes		PLS-MGA
R–SE	0.50	0.54	13.22	13.08	–0.04	.42
R–LC	0.04	–0.18	0.54	2.41	0.22	.03*
R–SNSU	–0.13	–0.12	2.29	1.55	–0.01	.90
R–CV	–0.02	–0.01	0.22	0.15	–0.01	.91
SE–LC	–0.25	–0.08	4.46	0.94	–0.17	.08
SE–SNSU	–0.25	–0.23	4.39	2.91	–0.02	.85
SE–CV	–0.06	–0.23	0.61	3.36	0.17	.17
LC–SNSU	0.08	0.15	1.38	2.44	–0.07	.40
LC–CV	–0.05	0.10	0.64	1.44	–0.15	.16
SNSU–CV	0.17	0.13	2.06	1.88	0.03	.72

*R* Resilience, *SE* Self-Esteem, *LC* Lover-Centeredness, *SNSU* Social Networking Site Use, *CV* Cyberbullying Victimization, *ß* Standardized Beta. *N*(No) = 342, *N*(Yes) = 255

## Discussion

This study examined the mediating roles of self-esteem, lover-centeredness, and social networking site use in the relationship between resilience and cyberbullying victimization (H1). A negative relationship was found between resilience and cyberbullying victimization. However, contrary to H1.1, resilience did not significantly predict cyberbullying victimization. This contrasts with existing literature, which suggests that resilience negatively impacts cyberbullying victimization [63, 64]. Personal, environmental, and biological factors embedded within resilience [65] may influence this result. Some resilience characteristics may only activate when faced with specific stressors [66]. For instance, Clements et al. [67] emphasized that individual differences, such as intellectual disabilities and chronic conditions, may mediate the relationship between resilience and cyberbullying victimization.

### Mediating Role of Self-Esteem

Consistent with H1.2, self-esteem significantly mediated the relationship between resilience and cyberbullying victimization. This suggests that self-esteem serves as a protective factor, aligning with prior research. Recent large-scale syntheses, such as the umbrella review conducted by Kasturiratna et al. [10], have identified low resilience and self-esteem as two of the most consistent psychological predictors of cyberbullying victimization across diverse populations. Our results align with and extend this body of evidence by demonstrating the mediating function of self-esteem within a serial mediation model.

For example, Erbiçer et al. [68] found a negative relationship between cyberbullying victimization and self-esteem in a meta-analysis. Resilience, which is closely tied to dimensions of self-esteem such as academic performance [45], physical appearance [47], and social success [46], may enhance individuals’ ability to develop healthier coping mechanisms in response to cyberbullying victimization [69]. Resilience fosters positive adaptation and growth, which are integral to self-esteem. Studies highlight the critical link between resilience and self-esteem in mitigating the effects of cyberbullying victimization [41, 70]. For instance, Santos et al. [41] emphasized resilience’s protective role in preserving mental health amid cyberbullying victimization.

### Mediating Role of Lover-Centeredness

This study did not support H1.3, which hypothesized that lover-centeredness mediates the relationship between resilience and cyberbullying victimization. Neither resilience significantly predicted lover-centeredness (H1.3.a), nor did lover-centeredness significantly predict cyberbullying victimization (H1.3.b). These non-significant paths indicate that lover-centeredness does not serve as a direct mediator in this relationship within the current sample.

However, additional findings suggest that lover-centeredness may still play an indirect role in the broader mediation model. Self-esteem negatively affected lover-centeredness (H1.5), while lover-centeredness positively influenced social networking site use (H1.7), which in turn was associated with increased cyberbullying victimization. While these associations do not support a direct effect, they point to a possible indirect pathway through which lover-centeredness could be involved in the cyberbullying process, though such interpretations should be made with caution.

The absence of a significant direct effect from lover-centeredness to cyberbullying victimization may be partially explained by contextual variables such as participants’ relationship satisfaction. For example, Geng et al. [71] found that satisfying the need for relatedness negatively correlates with cyberbullying victimization. Similarly, Self-Determination Theory [72] posits that fulfilling the need for relatedness reduces self-harming behaviors. Supporting this, multi-group analysis (H2) revealed that resilience significantly predicted lover-centeredness only among participants currently in a romantic relationship. This suggests that the impact of lover-centeredness may be shaped by relationship status, as well as individual differences in self-esteem and social networking sites use. Future research should further explore the potential contextual moderators that may influence the expression and effects of lover-centeredness in digital relational settings.

### Mediating Role of Social Networking Site Use

The findings supported H1.4, indicating that social networking site use significantly mediates the relationship between resilience and cyberbullying victimization. Resilience negatively influenced social networking site use (H1.4.a), while excessive social networking site use increased the risk of cyberbullying victimization (H1.4.b). Previous research shows that increased social networking site use is associated with greater exposure to online environments, thereby heightening the risk of cyberbullying victimization [73, 74]. Resilience may mitigate this risk by reducing problematic social networking site use [75, 76].

### Serial Mediating Roles of Self-Esteem, Lover-Centeredness, and Social Networking Site Use

This study confirmed H1, demonstrating that self-esteem, lover-centeredness, and social networking site use jointly mediate the relationship between resilience and cyberbullying victimization. Resilient individuals tend to have higher self-esteem, which is linked to lower lover-centeredness. Lover-centeredness, in turn, positively influences social networking site use, which is associated with higher cyberbullying victimization. Theoretical explanations suggest that individuals with high self-esteem focus on themselves rather than on their lovers [77]. High self-esteem is also associated with emotionally fulfilling forms of love devoid of jealousy or dependence [78]. Social networking sites offer a platform for individuals with high lover-centeredness to connect with their lovers or maintain their presence, influencing all stages of romantic relationships, from initiation to termination [79, 80].

### Practical Implications

The findings suggest that enhancing resilience through psychoeducational programs can reduce cyberbullying victimization by increasing self-esteem. These programs could also address lover-centeredness and its relationship to problematic social networking site use, further decreasing cyberbullying victimization. Prevention strategies, particularly for at-risk groups, can be implemented in university or community counseling centers. Experimental studies can test the effectiveness of such programs across different age groups and contexts.

### Limitations and Future Directions

This study has several limitations. First, its cross-sectional design limits causal interpretations. While we examined directional pathways between psychological factors, social networking sites use, and cyberbullying victimization, it is also possible that these relationships are bidirectional. For instance, individuals who experience cyberbullying victimization may increase their social networking sites use or change how they engage with platforms. Future longitudinal research could provide deeper insights into the directionality and temporal dynamics of these associations, as well as the effects of mediating variables. Second, data were collected using self-report measures and convenience sampling, which may limit generalizability. Future studies should consider diverse data sources and representative samples. Third, the study was conducted in a patriarchal cultural context with high levels of gender inequality. Fourth, the sample was predominantly composed of young adult females, which may limit the generalizability of the findings—particularly for constructs such as lover-centeredness that could be influenced by gendered social norms and expectations. Future research should aim to include more gender-diverse samples to better capture potential differences in relational dynamics and experiences of cyberbullying. Future cross-cultural research could explore how cultural factors shape the relationship between lover-centeredness and cyberbullying victimization. Fifth, while the proposed serial mediation model was theoretically grounded, alternative model configurations—such as parallel mediation or different mediator sequences—were not tested. Future studies should consider comparing multiple model specifications to evaluate the robustness and explanatory power of different pathways. Finally, while this study included a wide age range, it predominantly involved emerging adults. Future research could examine these dynamics in different age groups and socioeconomic contexts.

## Conclusions

This study provides empirical evidence on the relationship between resilience and cyberbullying victimization, highlighting the mediating roles of self-esteem, lover-centeredness, and social networking site use. While resilience did not directly predict cyberbullying victimization, its effects became significant through these mediators. The findings emphasize the importance of addressing individual factors, such as self-esteem and social networking site use, in interventions aimed at reducing cyberbullying victimization.

## Data Availability

The corresponding author can provide the dataset analyzed for this study upon request.
